# IL-10 Mediated Immunomodulation Limits Subepithelial Fibrosis and Repairs Airway Epithelium in Rejecting Airway Allografts

**DOI:** 10.3390/cells10051248

**Published:** 2021-05-19

**Authors:** Mohammad Afzal Khan, Ghazi Abdulmalik Ashoor, Talal Shamma, Fatimah Alanazi, Abdullah Altuhami, Shadab Kazmi, Hala Abdalrahman Ahmed, Abdullah Mohammed Assiri, Dieter Clemens Broering

**Affiliations:** 1 Organ Transplant Centre of Excellence, King Faisal Specialist Hospital and Research Centre, Riyadh 12713, Saudi Arabia; tshamma@kfshrc.edu.sa (T.S.); 83fatma@windowslive.com (F.A.); abaltuhami@kfshrc.edu.sa (A.A.); skazmi@kfshrc.edu.sa (S.K.); dbroering@kfshrc.edu.sa (D.C.B.); 2 Department of Physiological Sciences, Alfaisal University, Riyadh 11533, Saudi Arabia; gashoor@alfaisal.edu; 3 Comparative Medicine Department, King Faisal Specialist Hospital and Research Centre, Riyadh 12713, Saudi Arabia; halbasheer@kfshrc.edu.sa (H.A.A.); assiri@kfshrc.edu.sa (A.M.A.); 4 College of Medicine, Alfaisal University, Riyadh 11533, Saudi Arabia

**Keywords:** interleukin-10, immunotolerance, subepithelial fibrosis, TSG-6

## Abstract

Interleukin-10 plays a vital role in maintaining peripheral immunotolerance and favors a regulatory immune milieu through the suppression of T effector cells. Inflammation-induced microvascular loss has been associated with airway epithelial injury, which is a key pathological source of graft malfunctioning and subepithelial fibrosis in rejecting allografts. The regulatory immune phase maneuvers alloimmune inflammation through various regulatory modulators, and thereby promotes graft microvascular repair and suppresses the progression of fibrosis after transplantation. The present study was designed to investigate the therapeutic impact of IL-10 on immunotolerance, in particular, the reparative microenvironment, which negates airway epithelial injury, and fibrosis in a mouse model of airway graft rejection. Here, we depleted and reconstituted IL-10, and serially monitored the phase of immunotolerance, graft microvasculature, inflammatory cytokines, airway epithelium, and subepithelial collagen in rejecting airway transplants. We demonstrated that the IL-10 depletion suppresses FOXP3^+^ Tregs, tumor necrosis factor-inducible gene 6 protein (TSG-6), graft microvasculature, and establishes a pro-inflammatory phase, which augments airway epithelial injury and subepithelial collagen deposition while the IL-10 reconstitution facilitates FOXP3^+^ Tregs, TSG-6 deposition, graft microvasculature, and thereby favors airway epithelial repair and subepithelial collagen suppression. These findings establish a potential reparative modulation of IL-10-associated immunotolerance on microvascular, epithelial, and fibrotic remodeling, which could provide a vital therapeutic option to rescue rejecting transplants in clinical settings.

## 1. Background

Microvascular injury and associated tissue remodeling during acute rejection are caused by a massive microvascular infiltration of CD4^+^ T cells and antibody-mediated complement activation on vascular endothelial cells [1,2,3,4,5,6,7,8], and there are no ongoing immunosuppressive regimens that sufficiently preserve functional microvasculature [3,9,10]. This leads to the progression of chronic lung rejection/bronchiolitis obliterans syndrome (BOS) as seen in clinical settings [7,11]. Microvascular reestablishment and repair during rejection is a promising new avenue to prevent acute and chronic rejection with regulatory T cell (Treg)-mediated immunosuppression [12,13,14,15,16,17,18,19,20]. The T effector cells (CD4^+^) are key players during alloimmune inflammation; however, the immune regulation is tightly regulated through Tregs and associated molecular regulators [21,22,23]. 

IL-10 is a crucial immunoregulatory cytokine, which is secreted mainly by Tregs, monocytes, Th2 cells, subsets of activated T cells, and B cells [24,25,26]. The Treg-secreted IL-10 plays a vital role in preserving immunotolerance as well as the maintenance of FOXP3 expression, stability, and associated regulatory mediators, which help to counter inflammation, tissue repair, and antifibrotic events [24,27,28]. Besides, IL-10 gene expression further suggests the involvement of IL-10 as a potential molecular mediator to boost microvascular restoration [29,30,31,32,33,34,35]. IL-10 restrains inflammation through cell proliferation, differentiation and therefore suppresses major pro-inflammatory cytokines such as IFN-γ, IL-2, IL-3, and TNF-α produced by Th1 cells, activated Th1 cells, mast cells, NK cells, endothelium, eosinophils, and macrophages [36,37]. These pro-inflammatory cytokines can be deleterious to the host, and thus IL-10 limits potential tissue damage during an allograft rejection [35,38]. The role of IL-10 in transplantation is poorly understood, and because of its anti-inflammatory, regenerative, antifibrotic, and vasculoprotective properties, as well as its association with Tregs, IL-10 could play a discrete role in protecting grafts against the development of airway fibrosis. Previous research has emphasized that microvascular injuries and tissue remodeling post-transplantation have been initiated by a massive graft infiltration of T effector cells, B cells, and complement cascade activation [12,39,40,41,42]. While the effects of IL-10 in transplants have been established [29,43,44], the therapeutic benefits of IL-10 on microvascular and tissue repair remain unclear. Herein, we aimed to investigate the effects of IL-10 on the state of immunotolerance, and the subsequent influence on graft microvascular and pathological restoration during allograft rejection.

## 2. Materials and Methods

### 2.1. Mice Strains

All mice strains used in this research study were originally sourced from the Jackson Laboratory (JAX, Bar Harbor, ME, USA), and maintained as an original colony in an animal research facility at King Faisal Specialist Hospital and Research Centre (KFSH&RC), Riyadh, Saudi Arabia. In brief, C57BL/6J (B6.H-2b) mice strains were used as graft donors for syngrafts and as recipients of all other allografts, while BALB/CJ (H-2d) strains were used as allogeneic transplant donors for C57BL/6J in all transplants (Table 1).

### 2.2. Experimental Planning

The use of orthotopic tracheal transplantation (OTT) as a model for alloimmune and microvascular rejection is well-established [1,3,6,12,39,40,45]. The whole research plan was conducted in 28 days, and all transplants were serially monitored at 9, 10, 12, 14, 28 days post-transplantation as explained in Table 1. The selection criteria of the individual days were based on the occurrence of specific molecular/pathological symptoms during transplantation [1]. As reported in earlier studies in this model, d4 is the first point of microvascular reestablishment; d6–8 is the point where tissue oxygenation/microvascular blood flow peaks; d9–10 is the point of acute rejection with maximum lymphocyte infiltration, low tissue oxygenation, and no blood flow between donor and recipients; allografts further pass through an extended phase of hypoxia/ischemia between d12 and d14, and d28 corresponds to the point where sub-epithelium denudes with a significant occurrence of tissue and fibrotic remodeling [40,42]. 

### 2.3. Surgical Procedure

The KFSH&RC Animal Care and Use Committee (ACUC) approved the experimental protocol adopted in this study (RAC No. 2140 036). Four–six-ring tracheal segments from CO_2_-euthanized donor mice (C57BL/6J or BALB/CJ) were dissected out and used as a tracheal graft in C57BL/6J recipient mice as reported in previous studies [3,12,39,40,41,42]. To surgically connect the trachea, C57BL/6J recipient mice were anesthetized with ketamine (100 mg/kg) and xylazine (20 mg/kg), and a short incision was made in the middle neck region, which allowed the division of strap muscles and opened the surgical site of the recipient in the laryngotracheal area. Finally, donor and recipient tracheas were connected with 10-0 non-absorbable sterile polyamide monofilament suture (AROSurgical, Newport Beach, CA, USA) and the overlying skin was closed with 5-0 non-absorbable sterile polyamide monofilament suture (AROSurgical, USA). All transplanted mice have given post-operative medications carprofen (dose 5 mg/kg × SC) and Zolecin (dose 100 mg/kg × SC) and monitored for any respiratory distress in the first 24 h. In case of any respiratory distress, transplanted mice were immediately euthanized by CO_2_ as per ACUC protocols.

### 2.4. IL-10 Depletion (−) and Reconstitution (+)

To deplete IL-10, transplanted mice were given i.p. injections (250 μg/day) of anti-mouse IL-10 (Clone JES5-2A5) from Bio X Cell, Lebanon, NH, USA. To achieve maximum IL-10 depletion, all transplanted mice were injected for at least 2 weeks after the day of transplantation [46]. However, to reconstitute IL-10, transplants were intravenously injected with 4 μg of recombinant IL-10 (PeproTech, London, UK) in 100 μL of sterile PBS or with vehicle alone on days 3, 5, 7, and 9 post-transplantation [47]. An ELISA was run to confirm the serum levels of IL-10 post depletion and reconstitution experiments to validate the systemic IL-10 modulation. Based on our previous studies, we examined transplanted grafts for four weeks on selected day points to precisely demonstrate the effects of IL-10 on the discrete balance of proinflammatory and immunotolerance states on various graft pathological parameters during rejection.

### 2.5. Analysis of Regulatory T Cells

To analyze the peripheral T lymphocytes for CD4^+^ and FOXP3^+^ expression, blood samples were collected (BD-vacutainers) and a lymphocyte buffy coat was separated through the Histopaque gradient procedure as described in [12,39,40]. The mouse Treg specific markers were stained with APC-conjugated anti-mouse CD4^+^ (Clone RM4-5 RUO, BD Pharmingen) and PE-conjugated FOXP3^+^ (Clone MF23 RUO, BD Pharmingen) respectively as recommended by BD Pharmingen assay, which specifically sorts the CD4^+^FOXP3^+^ Treg subpopulation from mixed lymphocytes. Data were recorded at the flow rate of 14 µL/min and a minimum of 500,000 events were collected on the B6 Accuri flow cytometer and further analyzed through BD Accuri C6 integrated software [39,40].

Next, we further evaluated the extent of tissue-specific grafts infiltrating Tregs expression during rejection. Additionally, we immunostained graft depositions of TSG-6, which plays a supportive role in augmenting Tregs [48]. Transplants were harvested in Tissue-Tek O.C.T. medium (Sakura Finetek, CA, USA). Next, frozen samples were sliced into 5 μm-thick sections using a cryostat (Cryo 3, Sakura Fineteck, CA USA) and mounted on SuperFrost Plus slides (Fisher Scientific, Pittsburgh, PA, USA) for immunofluorescence staining. Slides were processed in methanol/acetone (1:1) and incubated with 10% donkey serum for 30 min and then incubated for 1 h with either rat anti-mouse CD4 (BD biosciences, San Jose, CA, USA), rabbit anti-mouse FOXP3 (Abcam, Cambridge, MA, USA), or anti-mouse TSG-6 primary antibodies (R & D Systems, Minneapolis, MN, USA). The slides were then washed with PBS, and sections were further incubated for 1 h with Alexa Fluor 488 donkey anti-rat (Jackson Immuno Research, West Grove, PA, USA), Alexa 647 donkey anti-rabbit (Jackson Immuno Research, USA), and Alexa 647 donkey anti-mouse (Jackson ImmunoResearch, USA) secondary antibodies. After incubation, sections were washed and mounted in Vectashield mounting medium (Vector Laboratories, Burlingame, CA, USA). Immunofluorescence image acquisition was performed with the EVOS FL auto cell imaging system (Life Technologies, Carlsbad, CA, USA), and the percentage of co-localization was quantified through the mean integrated fluorescence intensity of Alexa 488 to detect CD4 and Alexa 647 to detect FOXP3/or TSG-6 expression in separate graft samples per treatment group using ImageJ software [6,12,39,40]. 

### 2.6. Analysis of Serum Cytokines

Quantitative analysis of serum cytokines was performed by Milliplex MAP Mouse Th17 Magnetic Bead (Cat # MTH17MAG-47K). The serum was separated from blood after spinning at 1200 RCF for 10 min and stored at −80 °C for further use [12,39,40]. Quantitative estimation of serum cytokines was performed as suggested by manufacturer-approved protocol through antibody-linked magnetic beads on a 96-well plate. Plates were prepared with biotinylated detection antibodies and recorded on the Luminex 200 instrument in triplicates, as directed by the manufacturer’s instructions.

### 2.7. Analysis of Graft Blood Flow, Oxygenation, and Microvasculature

The tissue oxygen content (tpO2 mmHg) and blood flow (blood perfusion units (BPUs)) in transplants were measured in real-time by OxyLite/OxyFlo combined sensors (model NX-BF/OF/E, Oxford Optronix, Milton, UK) as originally described [41,42]. Transplanted mice were anesthetized and the airway grafts were surgically exposed, and finally, the sensors were inserted through 27 G needles to touch the airway epithelium for oxygen and blood flow measurement. Further, to confirm the functional donor-to-recipient graft microvasculature, all grafts were examined through lectin binding perfusion assay to demonstrate functional microvasculature between donor and recipient graft [3,12,40]. To test donor-recipient microvasculature, transplanted mice were intravenously injected with FITC-conjugated Lycopersicon esculentum (50 μL of 1 mg/mL) lectin under anesthetized mice, and after 5 min of lectin circulation, vasculature was washed with 1% PFA (paraformaldehyde), and grafts were harvested and incubated in 1% PFA at 4 °C for 10 min. Next, the grafts were mounted and examined through fluorescence microscopy (EVOS imaging system, Life Technologies, USA) [39,40].

### 2.8. Analysis of Graft Epithelium and Collagen Deposition

Pathological changes in graft epithelium and subepithelial collagen in IL-10 (−), IL-10 (+), and untreated control allografts were evaluated by H&E and trichrome staining as described in [3,6,12,39,40,49]. Harvested and Tissue-Tek O.C.T. medium (Sakura Finetek, CA, USA) processed graft sections on SuperFrost Plus slides (Fisher Scientific, Pittsburgh, PA, USA) were stained by H&E and trichrome to detect any pathological and structural perturbations in airway epithelium and subepithelial collagen deposition. Image acquisition was performed by capturing random high-powered fields per slide on the EVOS imaging system, Life Technologies, USA, and semiquantitative analysis was performed using the ImageJ program [39,40].

### 2.9. Statistical Analysis

GraphPad™ Prism software was used for statistical analysis to evaluate different transplants over time. Differences between various groups at multiple time points were compared using two-way ANOVA with Bonferroni multiple comparisons for post hoc analyses, while the differences between individual time points were compared by 1-way ANOVA or two-tailed *t*-tests and a *p*-value < 0.05 was considered as significant.

## 3. Results

### 3.1. IL-10 Is Sufficient to Establish Immunotolerance

**Figure 1:**
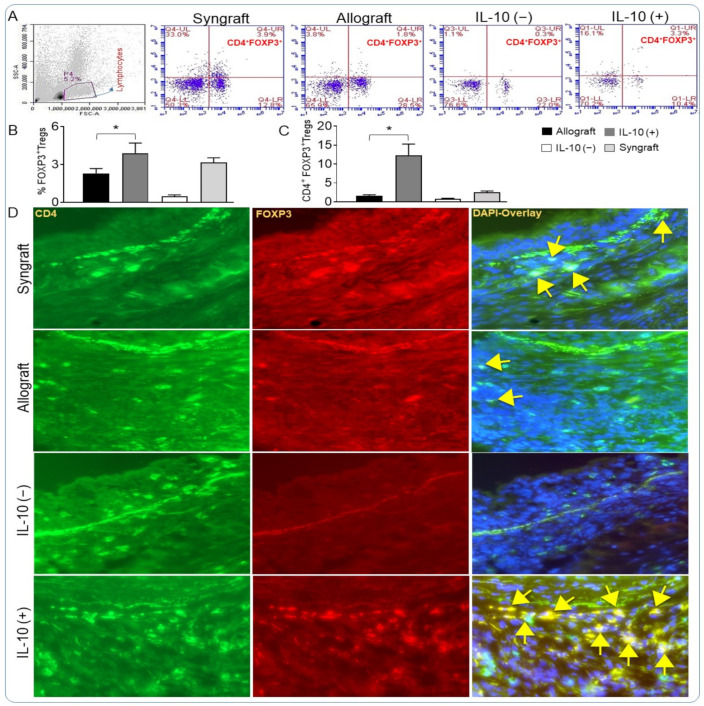
IL-10 is sufficient to establish immunotolerance in rejecting allograft. Flow cytometry analysis of Tregs from peripheral blood of transplants at d10 post-transplantation: (**A**,**B**) Percentage of gated lymphocytes and semi-quantitative analysis of FOXP3^+^ T cells in a given gated lymphocyte population. (**C**,**D**) Semi-quantitative analysis and immunofluorescent staining of CD4^+^FOXP3^+^ co-expression at d10 post-transplantation. Yellow arrows highlight CD4^+^FOXP3^+^ Treg cells. Data are presented as means with SE of 16 transplants/time point/experiment. * *p* < 0.05. Original magnification, ×40.

IL-10 is a key immunosuppressive cytokine and has previously been reported to subdue inflammation—this is a process that crucially modulates the physiological functioning of transplants, but knowledge of its immunoregulatory properties is limited. Here, we tested the pharmacological effects of IL-10 on peripheral and graft infiltrating CD4^+^FOXP3^+^ Tregs during transplantation. To check the peripheral Tregs during transplantation, PBMCs were isolated from syngrafts, untreated allografts, as well as IL-10 (−) and IL-10 (+) allografts at d10 post-transplantation and stained for CD4^+^ and FOXP3^+^ through flow cytometry. Our preliminary findings demonstrated that IL-10 (−) was associated with the suppression of peripheral CD4^+^FOXP3^+^ Tregs, while IL-10 (+) was associated with a substantial increase in CD4^+^FOXP3^+^ Tregs compared with both syngrafts and untreated allografts at d10 post-transplantation (Figure 1A,B). Because CD4^+^FOXP3^+^ Tregs have been associated with major regulatory activities and associated tissue repair, we next investigated whether the observed peripheral changes in Tregs were also associated with subepithelial deposition of CD4^+^FOXP3^+^ Tregs in the graft. We performed immunofluorescence staining of IL-10 (−), IL-10 (+), and control allografts. Immunofluorescence staining and image analyses demonstrated the downregulation of FOXP3^+^ Tregs, while IL-10 (+) allografts demonstrated a significant upregulation of CD4^+^FOXP3^+^ Tregs compared with syngrafts and control allograft at d10 post-transplantation (Figure 1C,D). These observations are consistent with the fact that the subepithelial deposition of Tregs is associated with the progression of the state of immunotolerance, which is vital to the graft repair post-transplantation [18,50,51].

### 3.2. IL-10 Is Sufficient to Augment TSG-6 Deposition

**Figure 2:**
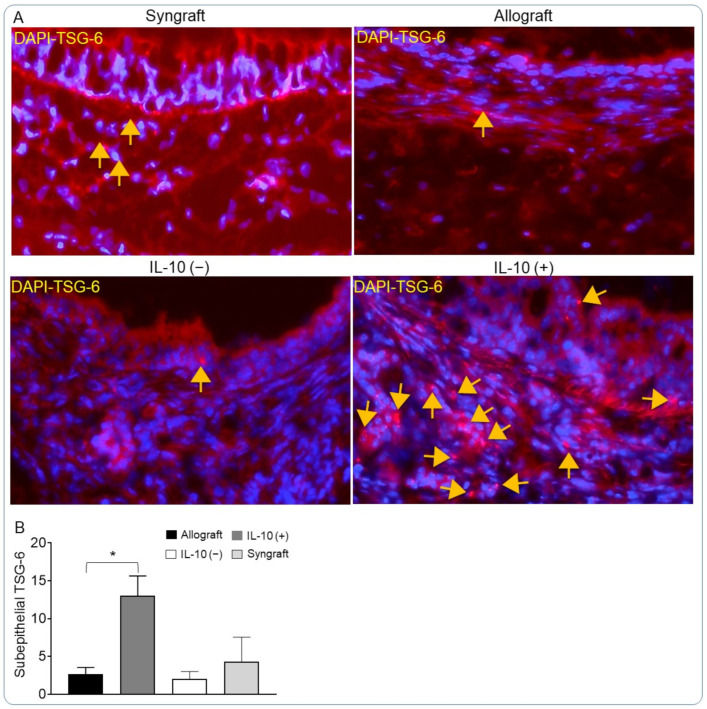
IL-10 is sufficient to augment TSG-6 deposition. (**A**) Immunofluorescent staining and (**B**) Semiquantitative analysis for subepithelial deposition of TSG-6 in control and IL-10 treated allografts at d10 post-transplantation. Data are presented as means with SE of 16 transplants/time point/experiment. * *p* < 0.05. Original magnification, ×40.

Tumor necrosis factor-inducible gene 6 protein (TSG-6) is an anti-inflammatory protein that is released during inflammation from different immune cells and has been associated with the suppression of IL-6, IFNγ, and TNFα, while increasing the generation of IL-10 and Tregs [48,52,53,54,55]. Here, we tested if IL-10 modulation affected the tissue deposition of TSG-6 during an inflammatory response. To examine this, we performed immunofluorescence staining of IL-10 (−), IL-10 (+), and control allografts for TSG-6 at d10 post-transplantation. Immunofluorescence imaging showed a substantial increase in the subepithelial deposition of TSG-6 in IL-10 (+) allografts compared with untreated control and IL-10 (−) allografts (Figure 2A,B). 

### 3.3. IL-10 Is Sufficient to Suppress Pro-Inflammatory Cytokines

**Figure 3:**
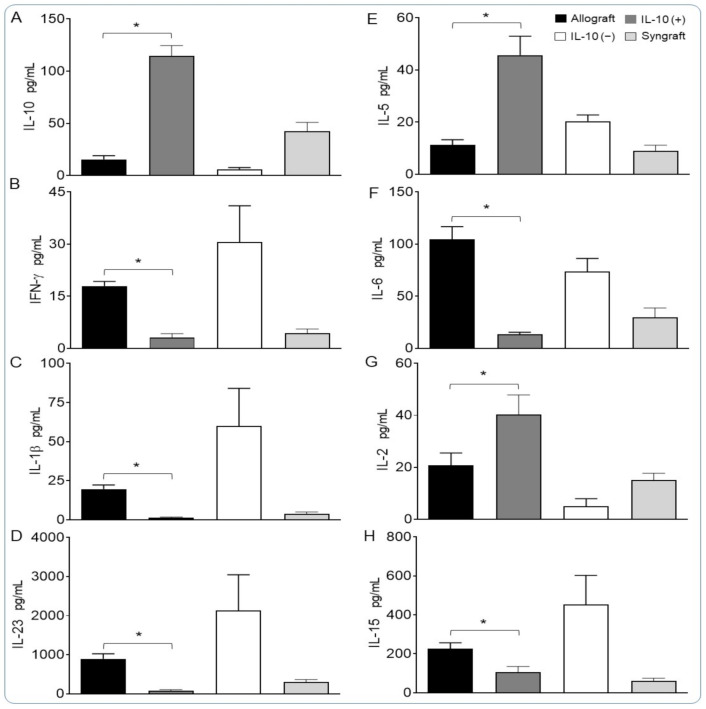
IL-10 is sufficient to suppress pro-inflammatory cytokines. Quantitative analysis (**A**) to validate the IL-10 depletion (−) and IL-10 reconstitution (+); (**B**–**H**) serum cytokines in control and IL-10-treated allografts at d10 post-transplantation. Data are presented as means with SE of 8–12 transplants/time point/experiment. * *p* < 0.05.

To quantify the serum levels of major proinflammatory cytokines, we collected serum at d10 post-transplantation and ran the ELISA through the LUMINEX multiplex assay. Data acquisition and analysis demonstrated a proof-of-concept that IL-10 (+) significantly downregulated proinflammatory cytokines (IFN-γ, IL-6, IL-1β, IL-23, IL-15) while IL-10 (−) favored the upregulation of corresponding proinflammatory cytokines in the serum at d10 post-transplantation, which is the main point of acute and microvascular rejection as seen previously (Figure 3A–H). However, IL-10 (+) allografts showed abrupt increases in IL-5 and IL-2 cytokines, which have been associated with supporting immunotolerance through Treg induction. These findings are evidence that enhanced regulatory activity in IL-10 (+) samples is pathologically correlated with the establishment of immunotolerance and an increase in the tissue expression of TSG-6, an anti-inflammatory molecule (Figure 3A–H).

### 3.4. IL-10 Is Sufficient to Restore Graft Oxygenation and Microvascular Blood Flow

**Figure 4:**
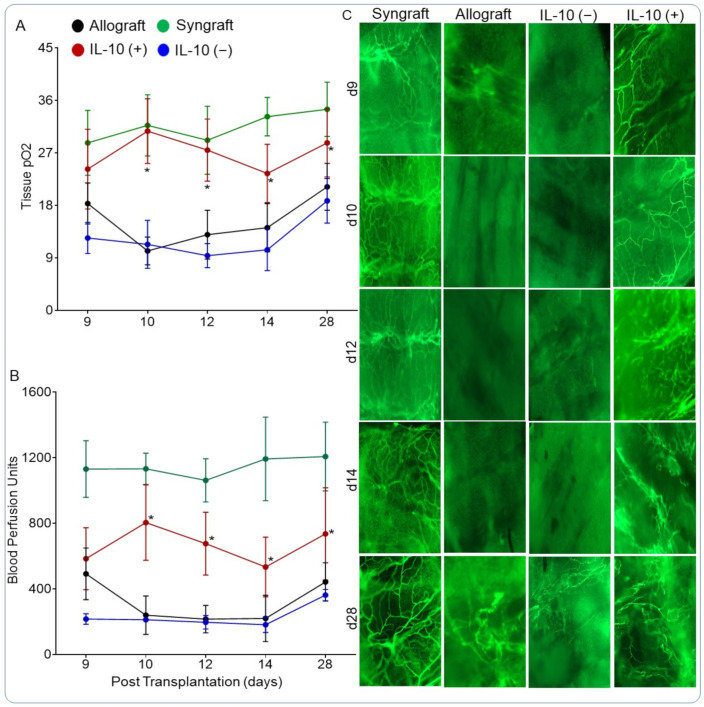
IL-10 is sufficient to restore graft oxygenation and microvascular blood flow. (**A**) Tissue pO_2_ (mean ± SE, mmHg) and (**B**) Blood perfusion units (mean ± SE, units) were plotted over different time points (d9–d28). (**C**) Lectin binding assay in control and IL-10-treated allografts at d9, d10, d12, d14, and d28 post-transplantation. Original magnification, ×20. Data are presented as means with SE of 16 transplants/time point/experiment. * *p* < 0.05.

IL-10 is a vital multifunctional cytokine that plays a crucial reparative role in graft microvascular health and thereby modulates hypoxic/ischemic state during rejection. While the effects of IL-10 blockade on immune suppression have already been reported [56], here we further delineate the effects of IL-10 modulation on donor-recipient graft microvasculature, graft oxygenation, and microvascular blood flow. To test this, we performed real-time measurements of tissue oxygenation and blood flow (measured in blood perfusion units, BPUs) in untreated control allografts, as well as IL-10 (−) and IL-10 (+) allografts from d9 to d28 post-transplantation (Figure 4A–C). Our results demonstrate that syngrafts remained oxygenated and fully perfused, while allografts passed through a long phase of hypoxia and ischemia from d9 to d14, and only showed a feeble microvascular rejuvenation at d28 post-transplantation. IL-10 (−) showed a similar trend in tissue oxygenation and blood flow, and passed through an extended period of hypoxia and ischemia from d9 to d14 and only showed a late but inadequate recovery in both tissue oxygenation and blood microvascular perfusion by d28 post-transplantation (Figure 4A,B). In contrast, IL-10 (+) allografts showed a significant increase in both tissue oxygenation and blood flow from d9 to d14 and consequently showed significant improvements in tissue oxygenation and blood flow at d28 post-transplantation as compared to IL-10 (−) and untreated control allografts (Figure 4A,B). Of note, a long phase of hypoxia and ischemia followed by a slow recovery in graft oxygenation and blood flow state in IL-10 (−) transplants as compared with IL-10 (+) allografts supported the notion that high levels of therapeutic IL-10 are vital to preserve and re-establish graft microvascular health (Figure 4A, B). Further, to investigate IL-10-mediated microvascular preservation, we investigated donor-to-recipient microvascular blood flow by lectin binding perfusion assay, which specifically detects a pattern of donor-to-recipient functional microvasculature during rejection [1,6,12,39,40,41,57]. Our results demonstrate that IL-10 (+) supported the reestablishment and preservation of donor-to-recipient functional microvasculature at d9, d10, d12, d14 (hypoxic/ischemic phase), and d28, while IL-10 (−) samples showed obliterated microvascular perfusion at corresponding day points as compared with untreated control allografts (Figure 4C). These findings support the notion that systemic IL-10 presence/absence directly affects the outcomes of alloimmune inflammation, and thereby modulates donor-to-recipient functional microvasculature and the progression of hypoxia/ischemia as seen in untreated allografts (Figure 4A–C). 

### 3.5. IL-10 Is Sufficient to Augment Airway Epithelial Repair and Subdue Fibrosis

**Figure 5:**
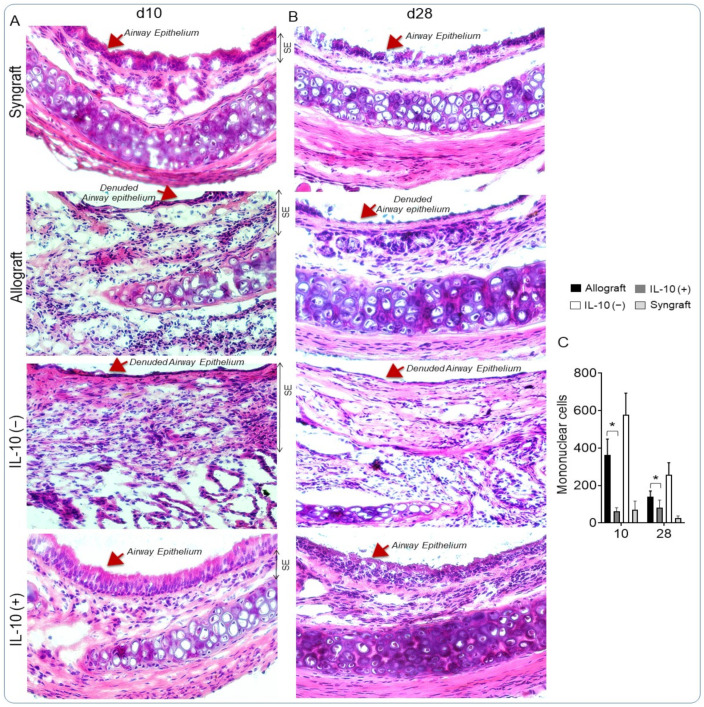
IL-10 is sufficient to augment airway epithelial repair. (**A**,**B**) H&E staining of graft transverse sections and (**C**) Subepithelial infiltrating mononuclear cells in control and IL-10-treated allografts at d10 and d28 post-transplantation. ‘SE’ designates subepithelial areas in the graft section. Data are shown as means with SE 16 transplants/time point/experiment. * *p* < 0.05. Original magnification, ×40.

**Figure 6:**
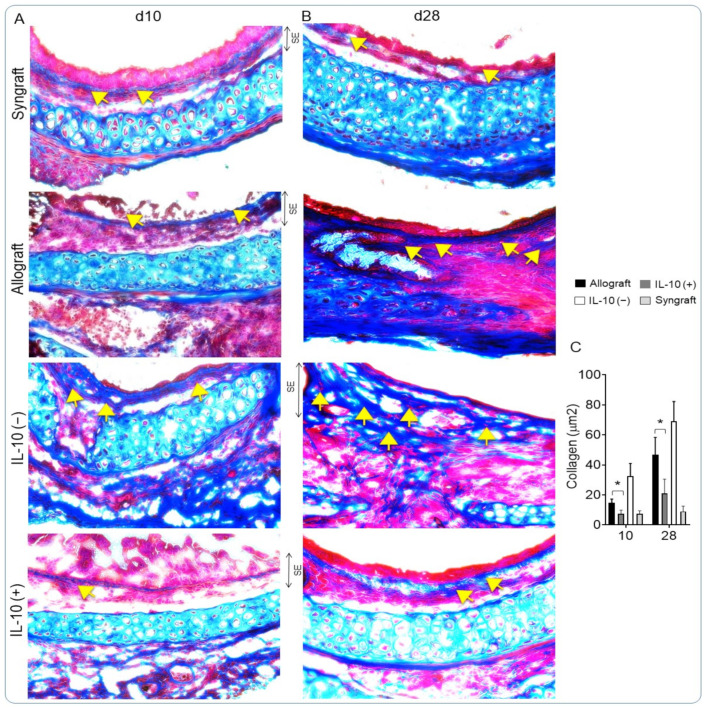
IL-10 is sufficient to suppress subepithelial fibrosis. (**A**,**B**) Collagen staining of graft transverse sections and (**C**) Semiquantitative analysis of subepithelial deposition of collagen in control and IL-10-treated allografts at d10 and d28 post-transplantation: Blue bands represent subepithelial collagen deposition, and semi-quantitative analysis of blue collagen bands was performed using the ImageJ program. ‘SE’ designates subepithelial areas in the graft section. Data are shown as means with SE 16 transplants/time point/experiment. * *p* < 0.05. Original magnification, ×40.

Alloimmune response and associated microvascular injuries are some of the key pathological changes that lead to airway epithelial injury and the progression of fibrosis post-transplantation [1,3,58,59]. Here, we examined airway epithelium and subepithelial collagen deposition in IL-10 (−) and IL-10 (+) allografts at d10 (point of acute rejection and microvascular loss) and d28 (point of microvascular reestablishment) post-transplantation. We stained samples with H&E and trichrome methods to demonstrate epithelial and subepithelial changes in all transplants. Morphological analysis showed the massive infiltration of subepithelial mononuclear cells followed by denuded airway epithelium in IL-10 (−) allografts. However, IL-10 (+) allografts showed significant downregulation of subepithelial mononuclear cells followed by a healthy airway epithelium at d10 and d28 compared with untreated control allografts (Figure 5A–C). Further, to examine the subepithelial antifibrotic effects of therapeutic doses of IL-10, we stained all samples with trichrome to map subepithelial collagen deposition. Morphometric analysis of collagen (blue bands) in the subepithelial region corresponded to fibrosis, as seen in untreated control and IL-10 (−) allografts, while IL-10 (+) allografts showed a significant drop in subepithelial collagen deposition. The histological analysis demonstrated crucial reparative and antifibrotic effects of IL-10 as compared with untreated control allografts, which is vital to long-term survival (Figure 6A–C). 

## 4. Discussion

IL-10 is an anti-inflammatory, anti-fibrotic immunoregulatory cytokine, which attenuates alloimmune response and displays pleiotropic effects on both innate and adaptive immunity [24,25,26,33,60,61,62]. Several experimental studies have demonstrated that IL-10 administration before transplantation facilitates tissue repair and graft survival [43,63,64,65,66]. 

Here, we hypothesized that IL-10 (+) is sufficient to upregulate FOXP3^+^ Tregs-mediated immunotolerance, suppress pro-inflammatory state, and thereby support microvascular repair and the suppression of subepithelial fibrosis through various regulatory mediators. Therefore, the purpose of this study was to further investigate the effects of IL-10 on immunotolerance and the inflammatory microenvironment, which are key to maintain airway tissue repair and suppress the progression of collagen during rejection. 

Although it is well known that IL-10 is a vital mediator of peripheral Tregs, the molecular impact of IL-10 on Tregs themselves is limited [28]. Our findings demonstrate that IL-10 (−) allografts showed significant downregulation of peripheral Tregs, graft deposition of TSG-6, increased proinflammatory cytokines, and passed through a comparatively longer hypoxic/ischemic phase, and favored persistent airway epithelial injury and subepithelial fibrosis. Meanwhile, IL-10 (+) allografts showed an upregulation of peripheral Tregs, graft deposition of TSG-6, increased IL-5 and IL-2, and passed through an extended phase of graft oxygenation and blood flow from d9 to d14 followed by significant microvascular recovery at d28 post-transplantation. Besides, IL-10 (+) favored airway epithelial repair and the suppression of subepithelial fibrosis, which confirmed that IL-10 is sufficient to modulate immunoregulatory and reparative effects to preserve graft health. Furthermore, persistent microvascular and airway injuries in IL-10 (−) allografts were highly correlated with tissue damage of normal airway structure, which was characterized by a massive subepithelial mononuclear cell infiltration as compared with untreated allografts. 

Microvascular injuries during an alloimmune response are crucial to the progression of various pathological alterations that require the coordination of various pro-inflammatory cells and molecular activities and have been a crucial hallmark of rejection in both small animal and clinical studies [1,3,7,8,41]. These epithelial injuries and the subsequent progression of fibrotic remodeling have been recognized as a key intermediate step that ultimately leads to obliterative airway disease, as seen in clinical settings [5,6,67,68,69,70,71,72]. Clinical data collected from obstructive bronchitis patients also demonstrated that the destruction of the epithelial cells during persistent alloimmune inflammation was initiated due to different cell and molecular inflammatory mediators in affected distal airways, which impeded the regeneration of airway epithelium and thereby progressed fibro-proliferation due to atypical tissue repair [68,73,74,75].

IL-10 is a vital cytokine for regulatory, reparative, and antifibrotic functions, but the mechanisms through which IL-10 augments these reparative and regulatory activities are likely the results of the pleiotropic effects of IL-10, as seen in various transplantation models [35,38,76,77,78,79,80]. These findings provide a proof-of-concept that IL-10 is vital for regenerative functions and associated with a proportional increase in anti-inflammatory TSG-6 protein, as well as the further upregulation of FOXP3^+^ Tregs, thereby supported the reestablishment of microvascular supply, tissue oxygenation, airway epithelial repair, and the suppression of collagen deposition in allografts. Moreover, an increase in Tregs in IL-10 (+)-treated samples further showed an increase in serum levels of both IL-5 and IL-2 cytokines, which has been reported to support Treg survival and expansion [81,82,83].

TSG-6 has been associated with the regulation of pro-inflammatory cytokines and the augmentation of tissue repair in various animal models while suppressing inflammatory reactions triggered by ischemia in the heart and thereby limiting the destruction of cardiomyocytes [48,52,53,54,55,84]. *TSG-6* gene inactivation has been associated with the upregulation of inflammatory immune reactions, while the over-expression of the TSG-6 gene has been associated with the downregulation of inflammatory reactions [85,86].

IL-10-mediated immune modulation and protection have been demonstrated in preclinical and clinical studies which highlighted the key immunosuppressive, protective, and antifibrotic roles of IL-10 in delaying the progression of fibrosis after transplantation [25,31,43,87]. IL-10 inhibits ischemia/reperfusion injury [78,88], prolongs allograft survival [61,87], and is essential for the action of Tregs mediating tolerance in different transplant models [28,62,89,90]. Besides, IL-10 is required for the immunoregulatory activity of Tregs as reported in the mouse model of skin transplantation, which demonstrated that blockade of IL-10 pathways abrogated immunoregulation mediated by CD25^+^ T cells [91]. Further studies in a rabbit model of skin transplantation demonstrated the key roles of IL-10 and Treg association during rejection, which concluded that IL-10 delayed the rejection time and thus prolonged the allograft survival time [43,61,87]. As reported earlier, IL-10 signaling affects the generation and function of Treg during tolerance through IL-10 receptors in Treg cells, and that signaling is required for Th17-mediated inflammation and subdues the toxic effects of inflammatory mediators during an immune reaction [24,90,92]. Both Treg and IL-10 have been associated with pathological angiogenesis, wound repair as reported in animal models of retinal neovascularization, left lung ischemia, type-2 diabetes, and airway allografts [12,38,39,40,76,77,79,93,94]. Of note, IL-10 is required for Tregs to mediate tolerance to alloantigens, as demonstrated in mouse skin transplants, which further highlights that IL-10 is required for Tregs’ immunosuppressive mechanism to preserve graft during extensive allograft rejection [91].

Here, we demonstrated a crucial relationship between IL-10, FOXP3^+^ Treg, TSG-6, graft microvascular health, and the progression of subepithelial fibrosis, which highlights the key therapeutic roles of IL-10 post-transplantation. These results demonstrate that IL-10 not only plays a pivotal role in establishing the phase of immunotolerance but also prevents the progression of collagen deposition in the graft. Furthermore, data also support the notion that IL-10 is a promising antifibrotic mediator, and therefore further studies are required to describe the specific therapeutic role of IL-10 in the progression of fibrosis. The delineation of this formerly uncharacterized mechanism supports the possibility that combination therapy of IL-10 with other immunosuppressive regimens would be an ideal option to reframe immunosuppressive therapies to halt tissue injuries and progression of fibrosis during chronic rejection. Therefore, IL-10 alone or in combination with other factors would be a potential therapeutic option to subdue the severe toxic effects of ongoing immunosuppressive regimens and to promote immune tolerance and graft repair in clinical settings.

## 5. Conclusions

Taken together, these findings highlight the key modulatory effects of IL-10 on immunotolerance and fibrosis, which further demonstrates a crucial strategy of IL-10-mediated immunotherapy to rescue rejecting allografts.

## Figures and Tables

**Table 1:** Experimental groups.

Donor	Recipient	Treatment Plan	Monitoring of Transplants
C57BL/6	C57BL/6	Vehicle-Treated Syngeneic Control	9, 10, 12, 14, 28
BALB/c	C57BL/6	Vehicle-Treated Allogeneic Control	9, 10, 12, 14, 28
BALB/c	C57BL/6	IL-10 Depletion	9, 10, 12, 14, 28
BALB/c	C57BL/6	IL-10 Reconstitution	9, 10, 12, 14, 28

Sample size (*n*) = 16 transplants/time point/experiment.

## Data Availability

The datasets used and/or analyzed during the current study are available from the corresponding author on request.

## Abbreviations

ANOVA: analysis of variance; BOS: bronchiolitis obliterans syndrome; BPU: blood perfusion units; d: days; FOXP3: forkhead box P3; FITC: fluorescein isothiocyanate; IL-10 (−): IL-10 depletion; IL-10 (+): IL-10 reconstitution; i.p.: intraperitoneal; MHC: major histocompatibility complex; OTT: orthotopic tracheal transplantation; PBS: phosphate-buffered saline; PBMC: peripheral blood mononuclear cell; PFA: paraformaldehyde; SC: Subcutaneous; tpO2: tissue oxygenation; TSG-6: tumor necrosis factor-stimulated gene 6; Tregs: regulatory T cells; Teffs: T effector cells; VEGF: vascular endothelial growth factor.
